# Facial Lesions as an Early Manifestation of Acute Leukemia in a Child With Down Syndrome: A Case Report

**DOI:** 10.1155/crpe/5239975

**Published:** 2025-06-10

**Authors:** Saeed Yousefian, Pedram Pirmoradian, Shirin Badihi

**Affiliations:** ^1^Department of Hematology Oncology, Isfahan University of Medical Sciences, Isfahan, Isfahan, Iran; ^2^Isfahan University of Medical Sciences, Isfahan, Isfahan, Iran

**Keywords:** acute myeloid leukemia, cellulitis, Down syndrome, pediatric

## Abstract

A 19-month-old female with Down syndrome presented with recurrent left cheek swelling and left eye involvement, initially diagnosed as fasciitis/myositis and periorbital cellulitis. Despite empiric antibiotics, symptoms persisted. A whole-body CT scan revealed prominent lymph nodes, and a biopsy of the buccal mass showed myeloid sarcoma. Bone marrow aspiration confirmed acute myeloid leukemia. Following chemotherapy, the patient's symptoms resolved. This case underscores that acute leukemia may manifest as facial swelling or periorbital cellulitis, highlighting the importance of considering extramedullary myelosarcoma in cases of persistent, unexplained soft tissue swelling that does not respond to standard treatments.

## 1. Introduction

Acute leukemia is characterized by the malignant proliferation of immature hematopoietic cells and disrupted hematopoiesis [1]. Myeloid sarcoma (MS), a rare extramedullary manifestation of hematologic malignancies, is composed of myeloid blasts and is most commonly associated with acute myeloid leukemia (AML), occurring in 2.5%–9.1% of cases [2, 3]. MS can develop at any age, with a slight male predominance, and most frequently affects the lymph nodes, skin, soft tissues, bone, and gastrointestinal tract [4]. Ocular involvement is reported in 35% of leukemia cases, primarily involving the retina [5].

Individuals with Down syndrome (DS) are at significantly higher risk of developing leukemia, particularly myeloid subtypes, with the majority presenting before age five [6–9]. Periorbital inflammation due to leukemia is rarely reported. Here, we present a rare case of MS in a child presenting with facial (cheek) and periorbital swelling that mimicked cellulitis but was ultimately diagnosed as MS.

## 2. Case Presentation

A 19-month-old female toddler, a native of Afghanistan with DS, presented with recurrent and progressive swelling of the left cheek, accompanied by involvement of the left eye. During her initial hospitalization, she exhibited swelling of the left cheek without a history of trauma or insect bites. A systemic physical examination revealed no abnormal findings. A complete blood count (CBC) showed no significant abnormalities: a white blood cell count of 5500 cells/μL (neutrophils: 62.0%, lymphocytes: 28.8%), hemoglobin of 10 g/dL, and a platelet count of 427,000 cells/μL. A multidetector computed tomography (CT) scan of the neck with contrast revealed fasciitis and myositis in the left masticator fossa. She was treated with antibiotics (piperacillin/tazobactam and vancomycin), and there was slight resolution of the condition.

**Figure 1:**
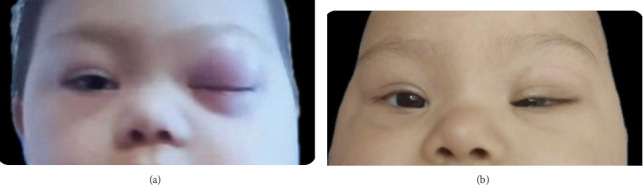
(a) Recurrence of cellulitis features in a patient, showing swelling of the left cheek and erythema around the left eye. The figure shows the child's presentation two weeks after initial treatment, with recurrence of cellulitis features as evidenced by swelling of the left cheek and erythema around the left eye. These findings suggest warranting further investigation and management. (b) Improvement of patient symptoms after chemotherapy. The figure shows the patient's condition after chemotherapy with significant improvement and regression of the previously observed swelling of the left cheek and erythema around the left eye.

Two weeks later, she returned with recurrent swelling and erythema at the corner of the left eye (Figure 1(a)). She had received outpatient treatment with clindamycin and cefixime but returned the next day with fever and a generalized rash. The patient was hospitalized with a clinical diagnosis of preseptal cellulitis and suspected orbital involvement. However, she was discharged against medical advice with a prescription for amoxicillin/clavulanate, along with recommendations for facial and cervical MRI and CT scans.

**Figure 2:**
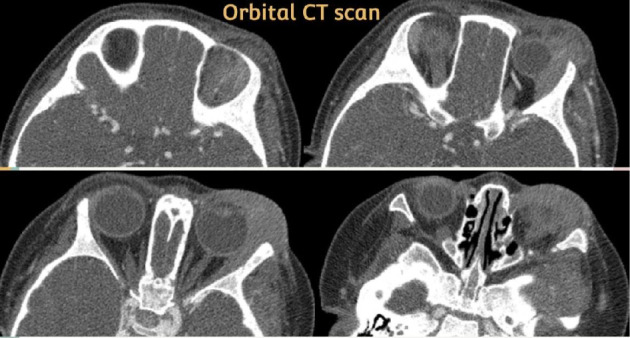
Orbital MDCT scan demonstrates left pre- and postseptal cellulitis, left-sided masticator and buccal space cellulitis, and left-sided otomastoiditis. Description: The Orbital MDCT scan with IV contrast shows that the right globe has a normal spheroid shape with a well-defined smooth contour. There is evidence of soft tissue infiltration and subcutaneous fat haziness in the left pre- and postseptal regions, left masticator fossa, and left buccal space, suggestive of cellulitis. Opacification is noted in the left middle ear cavity and mastoidal air cells, indicating otomastoiditis. These findings are suggestive of left pre- and postseptal cellulitis, left-sided cellulitis in the masticator and buccal spaces, bilateral cervical reactive lymph nodes, and left-sided otomastoiditis.

Two weeks later, she was brought back with worsening involvement of the left orbital and temporal areas, as well as snoring. Physical examination revealed significant findings, including tense swelling and purple discoloration of the left eyelids, along with swelling of the left cheek and temple. She was afebrile at the time. A repeat CBC, orbital CT scan (suggestive of left pre- and postseptal cellulitis, left-sided masticator and buccal space cellulitis, cervical reactive lymph nodes, and left-sided otomastoiditis) (Figure 2), chest CT scan (showing mildly prominent axillary lymph nodes), and abdominopelvic CT scan (showing a normal-sized liver, a spleen at the upper limit of normal size, and a few prominent mesenteric and para-aortic lymph nodes) were performed.

A biopsy of the periorbital/buccal mass was obtained, and immunohistochemistry revealed MS, with positive staining for CD45, CD34, and CD117 (focally positive), and a Ki-67 index of approximately 90%.

The patient was referred to an oncology and hematology hospital for further evaluation. A suspected diagnosis of acute leukemia prompted a bone marrow aspiration and flow cytometry, which confirmed AML. Trilineage hematopoiesis with about 20% blasts was observed. The results revealed a population in the blastic gate expressing CD34, CD117, dim CD13, CD33, HLA-DR, and aberrant CD7 (myeloblasts), constituting about 18% of all nucleated cells. Cytogenetic studies, including CBFB-MYH11 inv [10] qualitative analysis, FLT3-ITD mutation analysis, PML-RARA t [11, 12] qualitative analysis, and AML-ETO (RUNX1-RUNX1T1) t(8; 21) qualitative analysis, were performed but were negative for all.

Chemotherapy was initiated, consisting of pirarubicin (25 mg/m^2^/day for 2 days), cytarabine (100 mg/m^2^/day for 7 days), and etoposide (150 mg/m^2^/day for 3 days). No prophylaxis for central nervous system leukemia was administered [13]. The patient received five courses of chemotherapy. Signs and symptoms regressed after the first course, and the patient remained in remission for 30 months without any relapses (Figure 1(b)). Following this period of remission, relapse occurred.

## 3. Discussion

MS, also known as granulocytic sarcoma or chloroma, is characterized by the infiltration of immature myeloid cells or myeloblasts outside the bone marrow. Common symptoms include gingival hypertrophy, lymphadenopathy, and leukemia cutis, with the meninges, testes, and eyes being the most frequently affected organs in leukemia [5]. According to the French-American-British (FAB) classification, AML is divided into eight subtypes, with approximately 20% of cases belonging to the M4 and M5 subtypes [14]. These subtypes are more likely to infiltrate extramedullary sites, including the gingiva, skin, brain, spinal cord, and orbit. Extramedullary disease is associated with specific genetic alterations such as t(8; 21), inv [10], and 11q23 MLL mutations, and it can occur with or without concurrent bone marrow involvement [15].

Individuals with DS have a significantly higher risk of developing leukemia, with a cumulative risk of 2.1% by age 5% and 2.7% by age 30. Over half of these cases are myeloid, typically emerging before the age of five. Children with DS are approximately 500 times more likely to develop acute megakaryoblastic leukemia (AMKL, FAB M7) compared to the general pediatric population. DS-associated ML (DS-ML) has distinct features, including fewer cytogenetic abnormalities, lower initial white blood cell counts, and rare central nervous system involvement. GATA1 mutations, commonly found in DS-ML, are absent in patients over 4 years old [7–9]. While leukemia remission rates are high, with a 90% cure rate and a 70%–80% event-free survival (EFS) rate, individuals with DS remain at an elevated risk for both myeloid and lymphoid leukemia, which may occur independently in the same patient [16].

Periorbital and soft tissue cellulitis is characterized by eyelid and periorbital swelling, erythema, and fever. In one case, a 17-year-old patient presented with symptoms resembling periorbital cellulitis, including fever, erythema, and edema. Blood tests revealed anemia (Hemoglobin: 10.9 g/dL) and leukocytosis with immature blast cells. Bone marrow aspiration confirmed AML [17]. In another case, a 6-year-old patient presented with purpura, fever, and bilateral periorbital discoloration. Physical examination revealed hepatosplenomegaly, and blood tests showed leukocytosis with 70% myeloblasts containing Auer rods and anemia (Hemoglobin: 7 g/dL). Flow cytometry confirmed AML FAB-M2, with karyotyping revealing a t(8; 21) translocation. The patient underwent four courses of chemotherapy and remained in remission [11].

In our patient, no significant abnormalities were found in the CBC during the first hospitalization, and there were no signs of splenomegaly or fever. However, symptoms such as swelling and erythema of the left eye, recurrence, and lack of response to standard antibiotics, coupled with the known risk factor of DS, led to a reconsideration of the diagnosis. Granulocytic sarcoma can often be misdiagnosed as neuroblastoma, rhabdomyosarcoma, extramedullary hematopoiesis, eosinophilic sarcoma, periorbital cellulitis, or even abuse [10]. During the final hospitalization, CBC results showed leukopenia and anemia. The absence of sinus involvement on CT scans ruled out the typical spread of infection from the sinuses, and a whole-body CT scan revealed prominent lymph nodes. This case highlights that AML may initially present as facial swelling and periorbital inflammation, mimicking cellulitis. Diagnosis in such cases requires a thorough physical examination, blood cultures, laboratory tests, evaluation of potential infectious sources, and early hematology consultation in atypical presentations.

For DS-ML patients, treatment strategies aim to balance reduced intensity for standard-risk patients with intensified therapy for high-risk patients. Approximately 10%–15% of DS-ML cases fail to respond to current treatments, emphasizing the need for personalized therapeutic approaches [10].

## Data Availability

The data that support the findings of this study are available from the corresponding author upon reasonable request.
